# Impact of Endocrine Disruptors upon Non-Genetic Inheritance

**DOI:** 10.3390/ijms23063350

**Published:** 2022-03-20

**Authors:** Debbie Montjean, Anne-Sophie Neyroud, Marina G. Yefimova, Moncef Benkhalifa, Rosalie Cabry, Célia Ravel

**Affiliations:** 1 Fertilys Fertility Center, 1950 Rue Maurice-Gauvin #103, Laval, QC H7S 1Z5, Canada; benkhalifamoncef78@gmail.com; 2 CHU de Rennes, Département de Gynécologie Obstétrique et Reproduction Humaine-CECOS, Hôpital Sud, 16 Boulevard de Bulgarie, 35000 Rennes, France; anne-sophie.neyroud@chu-rennes.net; 3 Sechenov Institute of Evolutionary Physiology and Biochemistry, Russian Academy of Sciences, 194223 St-Petersburg, Russia; yefimova3@gmail.com; 4 Médecine et Biologie de la Reproduction, CECOS de Picardie, CHU Amiens, 80054 Amiens, France; cabry.rosalie@chu-amiens.fr; 5 UFR de Médecine, Université de Picardie Jules Verne, 80054 Amiens, France; 6 Peritox, Centre Universitaire de Recherche en Santé, Université de Picardie Jules Verne, 80054 Amiens, France; 7 CHU Rennes, Inserm, EHESP, Irset (Institut de Recherche en Santé, Environnement et Travail)—UMR_S 1085, University Rennes, 35000 Rennes, France

**Keywords:** endocrine-disrupting chemicals, transgenerational inheritance, non-genetic inheritance, epigenetics, embryo, sperm, oocyte

## Abstract

Similar to environmental factors, EDCs (endocrine-disrupting chemicals) can influence gene expression without modifying the DNA sequence. It is commonly accepted that the transgenerational inheritance of parentally acquired traits is conveyed by epigenetic alterations also known as “epimutations”. DNA methylation, acetylation, histone modification, RNA-mediated effects and extracellular vesicle effects are the mechanisms that have been described so far to be responsible for these epimutations. They may lead to the transgenerational inheritance of diverse phenotypes in the progeny when they occur in the germ cells of an affected individual. While EDC-induced health effects have dramatically increased over the past decade, limited effects on sperm epigenetics have been described. However, there has been a gain of interest in this issue in recent years. The gametes (sperm and oocyte) represent targets for EDCs and thus a route for environmentally induced changes over several generations. This review aims at providing an overview of the epigenetic mechanisms that might be implicated in this transgenerational inheritance.

## 1. Introduction

Endocrine-disrupting chemicals (EDCs) are exogenous chemicals or mixtures of chemicals interfering with any aspect of hormone action as defined by The Endocrine Society [1]. It is now established that more than 1000 products are considered as endocrine disruptors (https://endocrinedisruption.org, accessed on 26 January 2022). EDCs are environmental stressors able to activate or block hormone receptors. Their consequences can be deleterious, leading to cancer, malformations, or autistic disorders [2]. In particular, EDCs have been proved to have negative effects on male and female reproduction. Gametogenesis is a process tightly regulated by hormones, so is extremely sensitive to EDC. Androgens and estrogens play a key role in germ cell proliferation, development and survival. In the testis, minimal LH activity leading to the minimal production of testosterone by Leydig cells, first during “mini-puberty” in the postnatal period and then during puberty, is crucial to initiate and maintain complete spermatogenesis in adulthood [3]. Any anti-androgenic exposure may reduce plasma testosterone concentration, which might cause spermatogenesis dysfunction. Spermatogenesis is also modulated at every level by estrogen, from the hypothalamus–pituitary–gonadal axis to the testis cells constituted by Leydig, Sertoli and germ cells [4]. In the ovary, estrogen and progesterone are key steroid hormones in the complex regulation of female reproductive functions [5].

EDCs are able to dysregulate the endocrine pathways essential for hormonal homeostasis. Overdoses of testosterone administered to developing male ovine fetuses alter gene expression in the liver during adolescence, highlighting that prenatal androgen excess is a determinant of lifelong male metabolic health [6]. The whole life span of an individual may be affected by any disruption of homeostasis. The periconceptional time is a particularly sensitive period for environmental exposure, due to the major epigenetic processes that take place during gametogenesis and fertilization.

Both hormones and EDCs cause epigenetic changes either on DNA methylation, histone modifications or microRNA expression. EDCs interfere with the action of hormones and disrupt homeostasis. The timing of exposure is very important, particularly during early development, and any alteration in the germline during prenatal period will be transmitted to subsequent generations [7]. For example, the analysis of DNA methylation in potentially EDC-responsive genes revealed differential gene methylation within their promoter and/or gene body regions in the next generation [8].

If the classic notion of inheritance is based on the DNA molecule, new epigenetic actors are emerging [9]. Oocytes and spermatozoa are particular cells able to transmit information from one generation to the next. If an epigenome is altered, it will be transmitted to the embryonic stem cell and then to all adult cells. Moreover, the precursors of the gametes, named primordial germ cells (PGC) are prone to a fine and precise epigenetic regulation very sensitive to environmental factors [10]. Imprinted genes are epigenetic targets during gametogenesis able to retain and transfer environmental messages. Any gamete exposure to EDC has consequences on epigenetic markers and may interfere with the inheritance of specific features to the offspring. Several transgenerational studies demonstrate the transmission of phenotypes in the absence of direct exposure via the germline [11,12]. Moreover, several environmental toxicants were shown to promote the transgenerational transmission of increased disease susceptibility, infertility, general health conditions, the onset of disorders in the testis, prostate and kidney in males, and an increased incidence of obesity, polycystic ovaries, and reduced oocyte number in females [13]. Several players implicated in this non-genetic inheritance have been described so far: DNA and RNA methylation, histone modifications, non-coding RNAs and extracellular vesicles. The effects of EDCs in human gametes through epigenetic modifications are analyzed in this review and the potential transgenerational epigenetic effects are discussed.

### Non Genetic Inheritance Players

(a) Methylation

**Figure 1:**
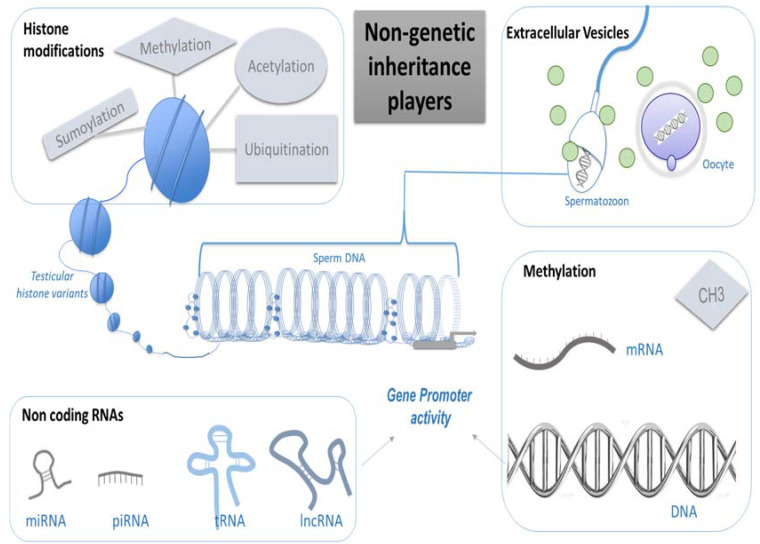
Schematic representation of the diversity of inheritance players, without modification of the DNA sequence. They are involved in the modification of gene expression. These modifications include histone sumoylation, methylation, acetylation, ubiquitination, miRNA, DNA methylation, RNA methylation and other non-coding RNAs. miRNA may be transported by extracellular vesicles. When the gene promotor is targeted by epigenetic changes, it directly affects gene expression. Such epigenetic modifications may affect both spermatozoa and oocytes. (miRNA: microRNA, piRNA: piwi-interacting RNA, tRNA: transfer RNA, lncRNA: long non-coding RNA, mRNA: messenger RNA).

DNA methylation regulates gene expression and genome activity without modification of the coding sequence (Figure 1). DNA methylation is technically easy to investigate and therefore the most documented epigenetic mark. DNA methylation involves methyl groups that are attached to a DNA molecule. The attachment of a methyl group to DNA is performed by DNA methyltransferase (DNMT) at a cytosine base just adjacent to a guanine residue, which results in 5-methylcytosine (5mC). Four human DNMTs have been characterized: DNMT1, DNMT2, DNMT3a and DNMT3b. De novo DNA methylation patterns are established early in development by DNMT3a and DNMT3b and maintained by DNMT1 [14]. When methylation affects a promoter region, it is associated with gene silencing. When it involves a transcribed region, it increases transcriptional activity. DNA methylation is essential for mammalian development. It is particularly involved in gene silencing, X-chromosome inactivation, parent-of-origin imprinting, and transposon silencing [15]. Genomic regions with a different DNA methylation status are called differentially methylated regions (DMRs). The epigenetic regulatory sites associated with DMRs may influence distal gene expression through non-coding RNA and are termed epigenetic control regions (ECR). According to Skinner’s definition, the altered epigenetic marks at a specific DNA site in response to an environmental factor to influence gene expression are termed an “Epimutation” [13]. The ability of an environmental factor to directly act and alter epigenetic processes to promote gene expression and phenotype alterations is defined as “Environmental Epigenetics”. Therefore, environmentally altered epigenetic sites that influence genome activity are epimutations [13]. Most of the epimutations reported in humans are somatic and erased in germ cells; however, cases of the secondary epimutation of a rare disease, present in three generations and maintained in germ cells, are described [16]. The DMRs identified have a low CpG density and exist in CpG deserts [17]. Other nucleic acid modifications have recently been described, namely DNA N6-methyladenine and RNA N6-methyladenosine, but the transmission of this epigenetic information across generations is still unclear [18].

(b) Histone Modifications

Histones serve to package and to organize DNA within the nucleus to form chromatin. In eukaryotes, the unit of organization of chromatin consists of a nucleosome with a protein core, which is an octamer containing two molecules each of histones H2A, H2B, H3, and H4. The DNA molecule is wrapped twice around a histone octamer. A linker histone H1 binds to the linker DNA, modulating the chromatin structure. Histone variants are characterized by a distinct protein sequence and a selection of designated chaperone systems and chromatin remodeling complexes that regulate their localization in the genome [19]. According to their composition of hydrophobic or hydrophilic amino acids, histone variants are prone to being associated with an open chromatin state by stabilizing nucleosomes, or a repressive chromatin state by destabilizing nucleosomes. For example, H2A.Z and H3.3 coincide with active gene expression, while macroH2A is found in transcriptionally inert chromatin [20]. In addition, histone variants can be enriched with specific post-translational modifications, which in turn can provide a scaffold for the recruitment of variant-specific interacting proteins to chromatin. These modifications include lysine acetylation, ubiquitination, sumoylation, lysine and arginine methylation, arginine citrullination, ADP-ribosylation, proline isomerization, serine/threonine/tyrosine phosphorylation and serine/threonine glycosylation, lysine biotinylation, monoaminylation of histone H3, glycation, lipidation, formylation or histone tail clipping [21]. All of these post-translational modifications of histones are epigenetic modulators of gene expression. Usually, histone acetylation favorizes gene transcription and is catalyzed by histone acetyltransferase (HAT), while methylation represses gene transcription [14]. The major targets of EDCs are nuclear hormone receptors, which bind steroid hormones and regulate the transcription of their target genes. Nuclear hormone receptors require coactivators linking the basal transcriptional machinery with the hormone receptors, and some of them possess HAT activity. These modifications can change the chromatin structure, modifying transcriptional cofactor recruitment and therefore gene expression. Some organotin compounds such as tributyltin (TBT) or triphenyltin (TPT) enhance the HAT activity of core histones in a dose-dependent way, and other EDCs such as monobutyltin or monophenyltin have no effect [22]. Fertility status in humans is correlated with histone H3 methylation changes in retained sperm histones [23]. Exposure to the organochlorine insecticide chlordecone increases prostatic epithelial neoplasia in F1 and F3 mice, associated with alterations in histone H3K4me3 [24]. Histone modifications are epigenetic marks that can be transmitted to the offspring [25].

(c) Non-coding RNAs

Non-coding RNA molecules are classified as long (>200 nucleotides) or small (<200 nucleotides). Long non-coding (lnc) RNAs are responsible for maintaining epigenetic memory through several mechanisms, including the regulation of DNA methylation, chromatin remodeling or histone modifications [26]. Like mRNAs, lncRNAs are transcribed by RNA polymerase II, and sometimes are also processed like mRNAs, have a 5mG cap, and are spliced and polyadenylated [27]. Several lncRNAs are reported to play a crucial role in stem cell maintenance and differentiation, involved in a variety of cancers, and important lncRNAs are known to play a functional role in spermatogenesis and male fertility [28]. The small RNAs (sncRNAs) class includes microRNAs (miRNAs), piwi-interacting RNAs (piRNAs), and endogenous-small interfering RNAs (endo-siRNAs), as well as other types of small non-coding RNAs derived from tRNAs, rRNAs, and small nucleolar RNAs (snoRNAs). tRNAs are of particular interest as a source of a heterogeneous class of small RNAs, tRNA-derived small RNAs (tsRNAs) [29]. tRNA biogenesis during post-testicular sperm maturation can regulate the expression of transcripts driven by endogenous retroelements modifying sperm epigenome in mammals [30]. tsRNAs represent a paternal epigenetic factor that may mediate the intergenerational inheritance of diet-induced metabolic disorders [31]. Environmental factors may cause primary DNA methylation changes, modifying the expression of adjacent ncRNAs and therefore affecting their target gene expression. However, environmental factors can also directly affect the production of ncRNAs, especially those large intergenic non-coding RNAs (lincRNAs) essential for sequence-specific DNA methylation and chromatin remodeling. Aberrant ncRNA production leads to altered DNA methylation patterns manifested as DMRs which, in turn, affect the expression of multiple mRNA genes located throughout the genome [26].

(d) Extracellular Vesicles

**Figure 2:**
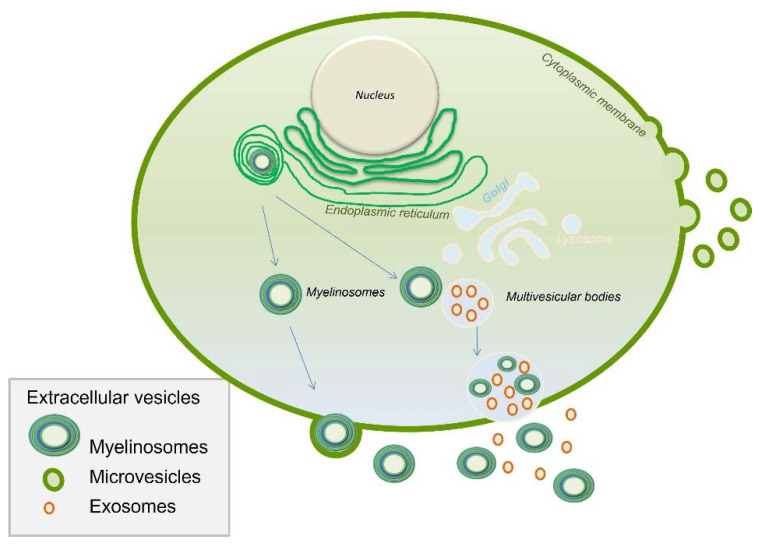
Schematic representation of the biogenesis and the diversity of extracellular vesicles (EVs). EVs are players in inheritance as they may transport diverse information to gametes and embryos.

Extracellular vesicles (EVs) are nanosized (<1000 nm), membrane-limited particles, heterogeneous in terms of size and content [32]. They transport multimolecular messages depending on the types of cells from which they are derived, in both physiological and pathological conditions [33]. Since they are present in body fluids, EV releases could serve as biomarkers [34]. Interestingly, EVs were proved to deliver a signal to recipient cells [35]. In the male genital tract, three subtypes of EVs are described: myelinosomes, exosomes and microvesicles (Figure 2). Myelinosomes and exosomes are issued from multivesicular bodies (MVBs) merging with the plasma membrane and released into the extracellular space, whereas microvesicles are formed by membrane shedding [36]. EV composition varies as a function of the secretion site, for example, the testis, prostate or epididymis [9]. In the female genital tract, EVs isolated from follicular and oviductal fluids exert a positive impact on embryo development in cattle [37]. EVs are involved in the regulation of genes implicated in follicular development, meiotic resumption, and ovulation, and act as a new means of communication in the ovarian follicle but also in embryo–maternal interactions [38]. Overall, EVs may play several roles in mammalian reproduction.

EVs are particularly enriched in ncRNAs, including miRNAs that target the mRNA, resulting in protein translation inhibition. These EVs contain RNAs that can be transferred to the gamete and most likely play a role in the development of the RNA profile during gametogenesis. It is not clear if EVs directly control gene expression, but they may participate in the epigenetic regulation of gene expression by transporting and delivering specific molecules [39]. The potential role of miRNAs in epigenetic regulation is strongly supported by these vesicle trafficking and protein carriers [40]. In particular, during the maturation of spermatozoa in the genital tract, specific epididymosomal EVs containing sRNAs are implicated in epigenetic inheritance from fathers to offspring (33). EVs also contain DNA, making possible genomic DNA exchange between cells [41]. This mechanism might be of relevance in the genital tract with the modification of the gamete environment, as EVs are sensitive to endocrine disruptors. For example, ubiquitous pollutants found in ambient air and diet exposure, such as polycyclic aromatic hydrocarbons (PAHs), increase the EV production and release in urine. Exposure to B[a]P (benzo[a]pyrene), can change the content of exosomes released by endothelial cells. EVs may be used as a sensor for the monitoring of exposure to B[a]P. B[a]P is considered to be a reference PAH and is an indicator of early cellular response prior to organ damage [42]. EVs are tiny intercellular messengers and the specificity of their content in terms of regulatory element such as RNA molecules makes them potential vehicles for environmental information transfer from the somatic cells to the germ cells [43]. It was also hypothesized that paternal extracellular vesicles could serve as vectors, delivering information not only to germ cells but also to the zygote after fertilization [44].

## 2. Endocrine-Disrupting Chemicals (EDCs) Transgenerational Impact

EDCs are found in lotions, cosmetics, soaps, perfumes, hair products, and feminine hygiene products. A recurrent exposure to low doses of EDCs may interfere with the endocrine metabolism. EDCs have been implicated in the development of cancers, mainly in hormone-dependent cancers such as prostate, testis, breast, endometrium or thyroid cancer [45]. It has been shown in a fish model that EDC-responsive genes are differentially methylated after exposure to EDCs. These genes are involved in steroidogenesis, prostaglandin synthesis, sexual development, DNA methylation, protein metabolism and synthesis, and cell signaling, but also in neurodevelopment [8].

### 2.1. Dioxin

Dioxins can occur through combustion or waste incineration, car traffic or cigarette smoking, but may also be caused by the manufacturing of paper, pesticides, herbicides, color metal or electronics. The most toxic chemical produced by humans is 2,3,7,8-tetrachlordibenzo-p-dioxin (TCDD) [46]. This results in the presence of a dioxin level higher than the tolerable weekly intake in the environment [47]. Dioxin inhibits estrogen-receptor-mediated gene transcription and modulates the expression or interact directly with steroid receptors [48]. TCDD promotes epigenetic transgenerational inheritance of disease and DNA methylation epimutations in sperm. In rats transgenerationally exposed to dioxin, transmitted kidney disease, pubertal abnormalities and ovarian disease/abnormality to their unexposed F3-generation descendants have been demonstrated [49]. Exposure to dioxin induces transgenerational effects on both female and male reproductive health. It decreases sex ratio (male/female), alters the onset of puberty and impairs both male and female fertility [50]. In males, TCDD exposure alters the steroidogenic gene expression in fetal and neonatal testes by reducing pituitary LH production, and reduces the expression of the cholesterol biosynthesis pathway genes in fetal testis, followed by decreased testosterone production [50]. In females, TCDD exposure in utero affects estradiol, FSH, and AMH levels. It impairs follicular development and leads to premature ovarian failure by involving mRNA expression, leading to the downregulation of the imprinted genes insulin-like growth factor 2 (Igf2) and H19, and the upregulation of Amh and Amhr2 [50]. The ancestral exposure of a gestating female to dioxin promotes an altered fetal gonadal development and epigenetic reprogramming of the germline that then transmits the altered epigenome to subsequent generations to contribute to the development of these ovarian diseases transgenerationally. This was confirmed by an exceptional human model: A high human exposure level to TCDD occurred in 1976 in an explosion at a chemical factory in Seveso (Italy), exposing nearby residents, which was associated with decreased fertility in Seveso mothers and potentially in their daughters exposed in utero. Ovarian diseases are found transgenerationally in the F3 generation, presenting primordial follicle loss or polycystic ovarian disease [46].

### 2.2. Diethylstilbestrol

Diethylstilbestrol (DES) is a potent estrogen compound that has been used for miscarriage prevention until the 70’s. Multigenerational effects of in utero exposure to this molecule have been comprehensively described [51]. DES binds to both estrogen receptors and progesterone receptors. The consequences of an historical accident where hormones were given to pregnant women without any previous test of their effect on the embryos were dramatically significant and affected up to three generations of children [51]. Daughters of women who were exposed prenatally to DES present an increased risk of menstrual irregularity and amenorrhea. Pregnancy outcomes were also affected with a higher incidence of preterm birth and a possibly increased risk of ectopic pregnancy. These observations support the hypothesis of epigenetic changes affecting primordial germ cells of the DES-exposed fetus. DES-exposed third generation women whose mothers had vaginal epithelial changes were more prone to show irregular menstrual periods. This phenomenon is a marker of early and high cumulative DES exposure [52]. Several case reports are highlighting an hypothetical implication in endometriosis [51], primary cell carcinoma of cervix [53] or androgen insensitivity syndrome [54] in children of grandmothers exposed. In utero exposure to DES was also suspected to contribute to the pathogenesis of psychiatric disorders since high prevalence of psychiatric disorders in two or three generations has been reported. This also put forward the possible multigenerational and transgenerational effects of DES exposure in neurodevelopment and psychiatric disorders [55].

### 2.3. Fungicides

Among the broad field of fungicides, the most studied according a transgenerational inheritance is vinclozolin, a fungicide used in agriculture with an anti-androgenic endocrine-disrupting activity. A transient embryonic exposure at a critical time during gonadal sex determination in rat, promotes male infertility associated with decreased spermatogenic capacity over three generations [11,56]. The molecular mechanism involved in epigenetic transgenerational inheritance requires hypo or hypermethylation of DNA in the germline to transmit the phenotype [56]. Prenatal exposure to vinclozolin caused sperm death and alteration of prostate function through the F3 generation. Sperm samples from three generation showed altered DNA methylation, ncRNA content and histone retention [12]. While in females, vinclozolin exposure was responsible for higher incidence of ovarian cysts and a dramatic reduction in oocytes through the F3 generation [57].

### 2.4. Organochlorine Pesticides

It is now well established that pesticides can modify the gene expression level by inducing different epigenetic changes, such as miRNA expression and DNA methylation status modulation [58]. DDT (1,1,1-trichloro-2,2-bis(4-chlorophenyl)ethane) and its metabolites (DDE and DDD) and methoxychlor (1,1,1-trichloro-2,2-bis(p-methoxyphenyl) ethane, MXC) are organochlorine pesticides [59]. Widely banned since the 1970s but still used in some countries, in particular for malaria control, DDT exposure persists due to global transportation. These molecules are persistent in the environment and bioaccumulate in lipid-rich organs [60]. The organochlorine pesticides and their metabolites possess estrogenic properties, impairing the activity of FSH and TSH receptors [60], with a negative impact on the reproductive system, as shown in animal models [61].

Although MXC has a low affinity for ERs, it has a modest endocrine activity and therefore is no longer used. Nevertheless, MXC primary metabolite, HPTE (2,2,-bis-(p-hydroxyphenyl)-1,1,1-trichloroethane), has a high affinity for ERs and is widely used as a model estrogenic EDC. DDT exposure induces an epigenetic transgenerational inheritance of sperm epimutations by the alteration of epigenetic processes, including DNA methylation, non-coding RNA (ncRNA) and histone retention. The most ncRNA-altered classes were piRNA and small tRNA. Histone replacement by protamines occurs during spermiogenesis, where transcriptional programs that lead to sperm specialization and sperm epigenome establishment are codependent mechanisms that have a direct role in the histone replacement and retention processes in the mammal’s sperm [62]. Histone retention is altered after exposure to DDT, since a large number of new retention sites were found in the sperm of exposed males, observed in a transgenerational manner [63]. In addition, females are also affected after exposure to DTT and MXC. Indeed, dramatic impacts of DTT and MXC were thoughtfully described in animal models and concerned female fertility, ovarian function, and implantation [64].

### 2.5. Bisphenol A

Bisphenols are found in domestic products and may be absorbed by oral and dermal routes. Bisphenol A (BPA) is a nonsteroidal estrogen and is one of the industrial synthetic chemicals produced at the highest volume worldwide [60]. BPA has toxic effects on oocyte maturation and spindle formation, and alters granulosa cell steroidogenesis. BPA can indirectly (through miRNA level modulation) upregulate the expression of genes involved in vascularization and angiogenesis that are crucial for endometrium growth during the menstrual cycle and decidualization [65]. The exposure to BPA in doses relevant to human exposure was shown to affect oogenesis and human oocyte maturation in vitro [66]. BPA exposure was also shown to affect female reproductive function in mice in a transgenerational manner [67]. Social behavior in mice and modifications in the expression of neural genes such as oxytocin and vasopressin were observed after ancestral exposure to BPA [68]. Even below the U.S. Food and Drug Administration (FDA) no-observed-adverse-effect level (NOAEL), prenatal BPA exposure may have adverse effect, since it was reported to disturb the transcriptome of the neonate amygdala in females. This prenatal exposure affects the developing brain by interaction with estrogen, oxytocin, and vasopressin signaling pathways. This results in the alteration of signaling pathways that are critical for synaptic organization and transmission [69]. BPA affects the epigenetic landscape since it impacts DNA methylation, histone modification and miRNAs expression, leading to the alteration of several metabolic pathways [70]. A deregulation of cellular and extracellular miRNAs may also be implicated in BPA toxicity in the ovarian follicle. It has been shown that exposure to supraphysiological BPA levels changes the levels of specific EV-enriched miRNAs in conditioned media of primary granulosa cells associated with modification in the expression of their cellular target genes [71].

### 2.6. PolyChlorinated Biphenyls

Polychlorinated biphenyls (PCBs) are a group of synthetic chlorinated aryl hydrocarbons extensively used in industrial applications, such as dielectrics, hydraulic fluids, lubricants and plasticizers. They are resistant to biodegradation processes and were banned twenty years ago. However, human exposure persists, with a NOAEL of 6–9 mg/kg/day [60]. This contamination is related to production through modern manufacturing processes and leaching from old construction materials and hazardous waste sites [72]. PCB sulfates are derived from the metabolism of hydroxylated PCBs (OH-PCBs) [73]. PCBs can interact with steroid receptors or modulate their expression. Based on the in silico simulation of molecule interactions, it was concluded that PCBs were able to interfere with the reproductive process [48]. PCBs decreased the force and amplitude of oviductal motility and some of them also stimulated the synthesis of leukemia inhibitory factor (LIF). LIF is released by oviductal epithelial cells and is indispensable for embryo implantation in the endometrium [74].

### 2.7. Phtalates

**Figure 3:**
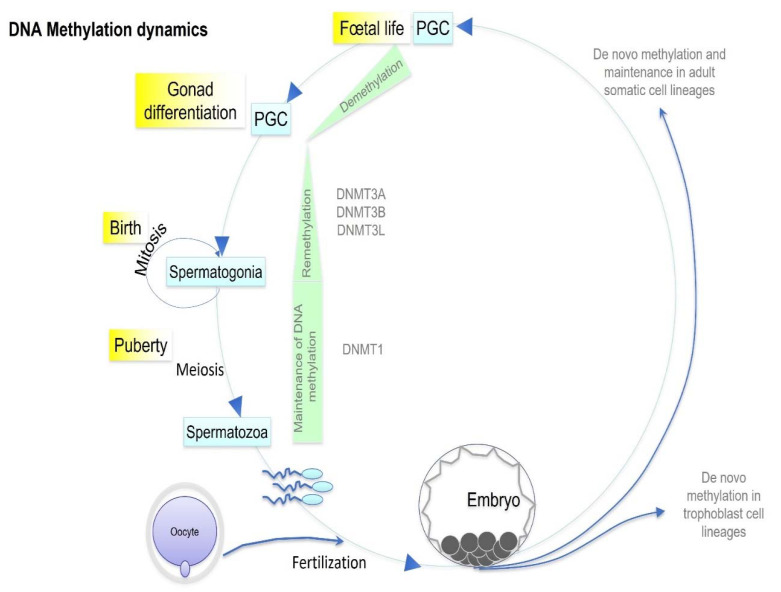
Schematic DNA methylation dynamics occurring during germ cell specification and embryo development. During mammal development, two waves of global demethylation occur: after fertilization and before PGC differentiation. Each step depicted in this figure may be a target for EDC with an impact on the DNA methylation profile, which may in turn affect the health of subsequent generations. PGC: primordial germ cells.

Phthalates and phthalate esters are compounds widely used in food processing and packaging. Di(2-ethylhexyl) phthalate (DEHP) is a plasticizer detected in a large variety of consumer products with a NOAEL of 20 mg/kg/day [60]. Owing to its anti-androgen activity, DEHP deregulates steroidogenesis and disrupts the reproductive system in both females and males in human and animal models. Mixtures of paternal urinary concentrations of DEHP metabolites are associated with higher rates of failure of couples’ infertility treatment [75]. Maternal DEHP exposure results in the DNA hypermethylation of promoters of spermatogenesis-related genes in fetal testicular germ cells in F1 mice. The hypermethylation of genes implicated in spermatogenesis (*Hist1h2ba*, *Sycp1*, and *Taf7l*) persisted from fetal testicular cells to adult spermatogonia, resulting in the downregulation of expression of these genes [76]. DEHP alters the sperm methylome as well as DNA methylation and gene expression in the developing embryo [77]. The fertility and reproduction of the third generation was shown to be disrupted in a sex-specific manner after ancestral prenatal exposure to DEHP. Indeed, males exhibited more severe adverse effect with decreased fertility, testicular steroidogenic capacity, and spermatogenesis. This observation suggested the involvement of the Y chromosome, which was supported by the results of testicular transcriptome analysis, showing an alteration of the expression of a number of Y chromosomal [78]. The impact of DEHP exposure on reproduction and social behavior is not only transgenerational but also dose-specific in both males and females [79]. In addition, the perinatal exposure of both juvenile and adult mice to DEHP induces sex- and tissue-specific DNA methylation alterations [80]. DEHP exposure during gestation increased DNMT3a and DNMT3b (Figure 3) expression in adult rat testes of F1 and in offspring, suggesting that epimutations may be a potential mechanism of DEHP-mediated testicular toxicity [81].

### 2.8. Perfluoroalkyl and Polyfluoroalkyl Substances

Perfluoroalkyl and polyfluoroalkyl substances (PFAS) are synthetic chemicals used for both household (e.g., shampoo, cookware) and industrial (e.g., insecticides) applications. They are insoluble in water and solvents and are bio-accumulated in the food chain with a long half-life. Nutrition is the main route of exposure in humans worldwide [82]. Perfluorooctane sulfonate (PFOS) and perfluorooctanoate (PFOA) are two fluorinated compounds that are widely used in industry and are commonly acknowledged as endocrine disruptors. PFAS have disruptive properties on the hypothalamic–pituitary–thyroid axis, which in turn have an adverse impact on neurodevelopment in utero and neonatal neuromaturation to adolescence and adulthood. Contamination is associated with reproductive toxicity, the depletion of the ovarian reserve, disruption in the earliest stage of folliculogenesis by altering oocyte development and the inhibition of steroidogenic enzyme activities. The potential mechanisms include the activation of peroxisome proliferator-activated receptor (PPAR) signaling pathways, the disruption of intercellular communication between oocytes and granulosa cells and the induction of oxidative stress [83]. In addition to PPAR signaling pathways, endocrine disruption may also be facilitated by acting directly on gene coding for the enzymes responsible for cholesterol transport and ovarian steroidogenesis, and a loss of kisspeptin signaling in the hypothalamus that can impact ovarian function [83].

Data from animal models about maternal PFOA exposure suggest that PFOA adversely impacts lactational efficiency, leading to offspring mortality, and altered mammary gene expression and mammary development [84]. PFOA and PFOS significantly reduced fecundity in a medaka model through different mode of actions [85].

### 2.9. Flame Retardants

Flame retardants are mostly volatile compounds that are included in objects of daily use such as furniture, clothing, toys, electronics, and plastics. They are released by these objects and thus detected in a large number of places, including house dust, food and water [86]. Three groups are described: organophosphate (OP) flame retardants (OPFRs), polybrominated diphenyl ethers (PBDEs) and novel brominated compounds. The utilization of the former has been largely banned since the mid-2000s due to their bioaccumulation in humans and wildlife, and their neurological and endocrine toxicity. As the use of polybrominated diphenyl ethers (PBDEs), and the entire class of organohalogen flame retardants, is declining, the use of OPFRs is increasing [87]. Men presenting higher concentrations of urinary OP metabolites, known to originate from flame-retardants, have aberrantly methylated sperm cells. Exposure to triphenyl phosphate is associated with hypermethylation at the GRB10 DMR, and tris(1,3-dichloro-2-propyl) phosphate exposure is associated with altered methylation at the MEG3 and H19 DMRs [88]. Tris(2-butoxyethyl) phosphate (TBOEP), which belongs to the group of non-halogenated OPs, shows endocrine disruption effects in daphnids. Exposure leads to significant differences in the transcription of genes involved in endocrine-mediated mechanisms such as reproduction and growth, indicating effects of parental exposure on offspring [89]. Tetrabromobisphenol A (TBBPA) is very popular and its exposure may result in neurotoxicity in zebra fish larval offspring [90]. Interestingly, it has been shown that the multiplicity of OPs in the human body is associated with increased DNA methylation aberrancies in sperm, compared with exposure to few OPs [88]

### 2.10. Polycyclic Aromatic Hydrocarbons

Polycyclic aromatic hydrocarbons (PAHs) are present in air pollutants and cigarette smoke components, and are environmental toxicants acting as chemicals disrupting endocrine regulation and reproductive toxicants. Benzo [a] pyrene (B[a]P) is one of the most important types of PAHs. PAHs are extensively metabolized by cytochrome P450 enzymes in humans and animals. The major metabolites of PAHs are monohydroxy-phenols (hydroxylated metabolites of polycyclic aromatic hydrocarbons (OH-PAHs)) and dihydrodiols, which are urinary biomarkers useful for the characterization of PAH exposure. Paternal preconceptional occupational exposure to PAHs was associated with increased risks of all childhood brain tumors [91]. In testis, PAHs interfere with gap junctional intercellular communication, which is critical for the normal development and function of testicular tissue [92]. Prenatal PAH exposure induces DNA methylation and alters gene expression in the Erα-mediated pathway across generations, suggesting that offspring consequences such as mammary cell proliferation also may occur in offspring as a result [93].

## 3. Epigenetic Inheritance

Epigenetic inheritance refers to the transmission of epigenetic marks to offspring [94]. It is intergenerational when the epigenetic marks are transmitted from one generation to the next. It is transgenerational when the information is transmitted from exposed grandparents to a grandchild, with an effect of the event observed in the third or fourth generation according the affected parent [95]. Transgenerational epigenetic inheritance has the potential to be adaptive, with major implications for heredity, breeding and evolution [96]. The origins of transgenerational germline epigenetic alterations have been shown to be throughout gametogenesis from the PGCs to the mature gametes [97]. The exposure of a gestating female to an environmental factor during pregnancy might directly affect offspring’s PGCs. An epigenetic change occurring in males can only modify his spermatozoa, affecting reliable nongenetic inheritance in the third generation. Multigenerational effects are defined when the affected generations are in direct exposure to EDCs [98].

Epigenetic information can be disrupted by environmental factors and could be inherited, leading to long-term adverse consequences affecting offspring health. For example, diet is an environmental factor modifying gene expression. Evidence of the epigenetic transgenerational inheritance of disease in humans is observed in Dutch and Swedish cohorts exposed to prenatal famine [99]. Exposure to famine in utero leads to higher rates of obesity, diabetes and mortality in offspring, related to epigenetic silencing [100]. Maternal undernutrition in goats altered the muscle fiber type in offspring, with the modification of several methylation marks [101]. An increased risk of mortality was described in grandchildren when grandparents had been exposed to famine. Health and behavior disorders were also detected at a higher incidence in the offspring when the mother had been exposed to famine [99]. Oppositely, in humans, increased maternal BMI was associated with an alteration in DNA methylation landscape in offspring both in neonates and in later childhood [102]. This observation suggests that differential gene methylation and thus gene regulation is highly dependent on the maternal environment [103]. In another study, a transgenerational kidney disease was observed in male and female third-generation descendants of gestating females exposed to methoxychlor, showing that methoxychlor could transmit kidney diseases and obesity through the female germline [104].

It is difficult for human studies to highlight a clear connection from exposure in an ancestor leading to molecular changes in germ cells, driving a specific phenotype in descendants. However, associations have been made between several aspects involving epidemiological, epigenetic, and genetic approaches [105]. An example of particular interest is the development of polycystic ovary syndrome (PCOS), closely related to epigenetic mechanisms. It is a heritable affection and common metabolic and reproductive phenotypes were described in the parents of PCOS women [106]. This syndrome is not without any consequences on the lifetime risk of comorbidities, since it dramatically increases the chances of developing type 2 diabetes mellitus, psychiatric disorders and gynecological cancers. Hyperandrogenemia is one of the features of PCOS, which persists throughout reproductive life and after menopause [107]. Several dysfunctions, such as reproductive, metabolic or psychiatric dysfunctions, are correlated with a high level of circulating androgens. It was suggested that hormonal dysregulation of the maternal uterine environment led to epigenetic and developmental programming, resulting in the pathogenesis of PCOS. The exposure of mothers is the main cause for the observed transgenerational effects of androgen exposure, which are passed on for up to three generations [108]. This epigenetic inheritance is supported by the observation of family members that also suffer from an increased risk of developing PCOS-associated reproductive and metabolic disorders [109]. It was also hypothesized that an alteration of androgens or anti-Mullerian hormone (AMH) levels during pregnancy could be responsible for PCOS in female newborns [109]. In addition, the expression of ovarian genes may be altered in the third generation after ancestral prenatal AMH exposure [110]. Indeed, some phenotypes such as reduced sensitivity to thyroid hormone, mortality, type II diabetes, asthma, spina bifida, metabolic syndrome, and genitourinary abnormalities in great grand-children may be a consequence of ancestral exposure and inherited in a non-genetic manner [105].

A transgenerational transmission of increased incidence of disease is described through the male germline after exposure with vinclozolin [11] or through the female germline after methoxychlor exposure [104]. DDT exposure determined that male obesity was transmitted through the female germline and female obesity through the male germline. The female germline transmission of environmentally induced epigenetic transgenerational phenotypes appears to be as stable as male germline transmission, and the combination of both paternal and maternal alleles is needed to transmit certain diseases, such as testis disease, to the male offspring [111]. The paternal inheritance of psychological post-stress effects has been reported, showing that the inheritance of “epigenetic memory” produced offspring with the potential to be adapted to the environmental challenges that their parents experienced, with major implications for heredity and evolution [112].

## 4. After-Effects of EDCs Exposure on Germ Cells

EDCs are an important part of our environment. Exposure to EDCs results in epigenetic modifications in cells. Germ cells are particularly vulnerable to EDC exposure. The epigenetic markers most studied are DNA methylation, the modification of histones, ncRNA and EVs (Figure 1).

### 4.1. Epigenetic Sperm Modifications

Sperm cells have long been considered as delivering the paternal haploid genome and then the genetic information to the oocyte. However, spermatozoa also harbor epigenetic information that plays a remarkable role during offspring early development and long-term health. These multiple epigenetic marks make up the epigenome [113]. The blood–testis barrier reduces the potential for xenobiotic agents to alter the germline. However, electrolytes, very small polar molecules and some classes of lipophilic molecules have a high transfer potential to cross this barrier [43]. Epigenetic alterations occur during the development of male fetal germ cells, leading to spermatogenesis failure and the alteration of sperm parameters in adulthood [113].

The DNA methylation profile of male germ cells is significantly different from that of somatic cells and is stable throughout spermatogenesis and in mature sperm. Germ cell DNA demethylation and remethylation in a sex-specific manner occurs during fetal development and is maintained throughout the individual’s entire life [113]. Environmental factors can induce changes in DNA methylation markers in the germ line [114]. In humans, the exposure to chemotherapy, bariatric surgery, cannabis, flame retardants, mercury, polycyclic aromatic hydrocarbons and bisphenol A was found to induce sperm DNA methylation alterations [105]. It is interesting to note that the methylation profiling of PEG1/MEST-DMR and H19-DMR in sperm shows epimutations in H19-DMR and PEG1/MEST-DM in men with reduced sperm counts without consequences on the outcomes of assisted reproductive techniques [115]. A similar observation was made at the genome-wide scale and a significant positive association between sperm global DNA methylation level and sperm concentration was described [116,117]. Endocrine disruptors have been shown in mouse models to induce transmissible changes over several generations, altering the quality of spermatogenesis in adulthood [11]. These epigenetic markers are easily modifiable by exogenous factors such as bacterial infection [118]. It has been shown that changes in apparently harmless habits such as physical training can alter the sperm methylome [119]. Age-associated methylation has been reported, supporting a link with neuropsychiatric disorders such as autism spectrum disorder, schizophrenia, and bipolar disorder in the offspring born after advanced age males [120]. The paternal methylome was also shown to be sensitive to environmental factors with perturbations persisting for at least two subsequent generations [121].

In adulthood, during spermiogenesis, chromatin is reorganized in the male gamete. Most of the histones are replaced by protamines, allowing supercoiling and chromatin compaction, but up to 10% of histones are preserved in the sperm [113]. This protamine–histone–DNA organization during spermatogenesis is specific to the sperm epigenome, Thus, the paternal genome becomes transiently vulnerable to environmental hazards during this chromatin rearrangement process and any exogenous perturbation to the sperm epigenome may have serious impacts on subsequent offspring development [122]. A significant correlation between nonoccupational exposure urinary levels of 1-OHP, 1-OHPH and the methylation of sperm DNA imprinting genes suggests that sperm chromatin is sensitive to PAHs [123]. Alterations in histone retention appear to take part in the environmental induction of epigenetic transgenerational inheritance [124].

Large (mRNAs and lncRNAs) and small (sncRNAs) sperm-borne RNAs are delivered to the oocyte during main fertilization. The sperm of transgenerational males that were ancestrally exposed to DDT have differentially expressed lncRNAs. Paternally acquired characteristics may be transmitted to the offspring via sperm RNAs in the first post-fertilization mitotic divisions of the zygote [125]. miRNA and piRNA expression in sperm are affected by life factors such as endurance training, stress in childhood and cigarette smoking, with a potential impact on general health in subsequent generations [105], suggesting a high susceptibility to environmental factors.

Specific post-testicular sperm maturation occurs through the epididymal secretion of EVs [9]. Reproductive tract EVs transmit information regarding stress in the paternal environment to sperm, potentially altering fetal development. It has been shown that sperm incubated with EVs collected from stress-treated epididymal epithelial cells produced offspring with altered neurodevelopment and adult stress reactivity [126]. Proteomic and transcriptomic assessment of these EVs showed dramatic changes in protein and miRNA content long after stress treatment had ended [126]. These data confirm that EVs are a normal players in sperm maturation but also perform roles in the intergenerational transmission of paternal environmental exposure [127].

### 4.2. Epigenetic Oocyte Modifications

Oocytes are subjected to prolonged periods of arrest. There are critical periods wherein the oocyte undergoes nuclear and cytoplasmic maturation, where the translation of stored mRNAs, accumulated during the growth period, is crucial for meiotic maturation and subsequent embryogenesis. The maintenance of protein homeostasis is needed to achieve successful fertilization despite proteostasis being “reset” during embryogenesis [128]. Environmental exposure leads to an earlier age at menopause, premature ovarian failure and infertility by exhausting the oocyte pool and causing the depletion of follicular ovarian cells [129].

In utero exposure to a plastics mixture during the period of fetal gonadal sex determination promotes the epigenetic transgenerational inheritance of adult-onset disease resembling primary ovarian insufficiency and polycystic ovarian syndrome (PCOS) [57]. Plastic mixtures (BPA, DEHP, DBP) caused early-onset puberty compared with the control, and both the pesticide and plastic mixtures caused a significant decrease in the primordial follicle pool compared with the control in rats [130]. A gene network analysis of the transgenerationally altered granulosa cell transcriptome highlighted a set of potential regulatory genes associated with ovarian abnormalities [57]. The critical window of exposure to promote such changes in humans is 6–18 weeks of gestation [57].

Vinclozolin-lineage granulosa cells displayed significant transgenerational alteration in both the transcriptome and epigenome, bringing up a new paradigm for the etiology of ovarian disease [104]. Moreover, the oocytes, the embryos, the endometrium, and clinical outcomes in IVF are also adversely impacted by the exposure to various pesticides [131].

Significantly, more DMRs are found when gestating female rats are exposed to vinclozin, in F3 compared with F1 sperm, knowing that these animals were exposed during a period of PGCs deprogramming and subsequent reprogramming. Methylation patterns between generations depend on the period during which the F1 animals are exposed [132].

The transmission of environmentally induced epigenetic changes through the female germline appears to be stable, but a combination of both paternal and maternal alleles is needed to transmit testis disease to the male offspring [111]. Despite the importance of DNA methylation in the oocyte, knowledge of its perturbation in human oocytes remains very limited.

In the oogenesis and early development of mammals, the functional activity of chromatin is regulated by the unique epigenetic landscape created by post-replication DNA modifications, post-translational modifications of DNA-associated proteins and ATP-dependent nucleosome remodeling. In the oocyte, chromatin undergoes chromosome condensation, withstands double-stranded breaks during meiotic recombination, and survives a long meiotic arrest in mammals [133]. However, oogenesis does not appear to involve dramatic chromatin changes analogous to the protamine transition during spermiogenesis [134]. The maternal inheritance of certain histone variants is essential for embryonic viability and development [19]. Histone variants may “mark” imprinted regions of the inherited maternal genome in embryos. After fertilization, the protamine-bound sperm chromatin becomes decondensed into the zygote, and protamines are quickly replaced by the oocyte-specific H1, H1oo, leading to an enlarged sperm pronucleus [135]. Maternally histone variants may also be crucial for the initial stages of embryogenesis, especially to mediate the protamine-to-histone transition of the paternal genome and post-translational modifications of histones for zygotic genome activation. Despite these specialized chromatin requirements, very few chromatin exogenous alterations have been described for mammalian oogenesis, unlike for spermatogenesis [134].

Among lncRNAs, Xist (X-inactive specific transcript) acts in the silencing of the X chromosome by modifying the structure of the chromatin and the factors interacting in chromosome X of mammalian females during development. Dicer1 is an important RNase III enzyme that processes pre-miRNA into the shorter miRNA duplex. Conditional knockout of Dicer in mice increases the degeneration of follicles and decreases ovulation rates. miRNAs are involved in ovarian folliculogenesis and granulosa cell physiology [136]. piRNAs are present in ovarian follicle cells and may suppress transposons in the ovarian somatic cells [137].

Concerning EVs’ implication in oocyte maturation, EVs derived from plasma promote cumulus expansion and oocyte maturation by enhancing Has2 and Ptgs2 mRNA expression in the cumulus–oocyte complex [138].

## 5. Embryo Development and Critical Window

Exposure during pregnancy to environmental factors increases the risk for disease development later in life [139]. Transgenerational effects of low doses of EDCs are mainly attributed to epigenetic changes, presumably at the level of the germ cell. PGCs are the precursors of gametes. During their migration to the genital ridge, PGC undergo an epigenetic reprogramming to establish methylation on imprinted genes in a sex-specific manner [140]. This epigenome resetting, including chromatin remodeling and global DNA demethylation, occurs within ∼4 weeks in human PGCs (131). A dramatic remodeling of constitutive heterochromatin occurs, which is essential for natural reprogramming at fertilization. In the paternal pronucleus, H3K9me3 is catalyzed by SUV39H2 (a histone lysine methyltransferase) after fertilization. De novo H3K9me3 is initially non-repressive for gene expression, but instead bookmarks promoters for compaction [141]. These early stages of development represent a critical window of vulnerability to the effects of EDCs, as any perturbation by exogenous compounds may alter PGC specification, migration, and differentiation. Several genetic factors have been identified, such as *Blimp1*, *Prdm14*, and *Tcfap2c*, *Lin28* and the microRNA *let-7* [142]. Blimp1 is a crucial regulator of PGC differentiation and prenatal exposure to vinclozolin, and induces a disequilibrium in the *Lin28/let-7/Blimp1* pathway in three successive generations of males mice [10]. Following fertilization, DNA methylation erasure occurs in the stem cells of the early developing embryos, before the global resetting of the methylation landscape in different somatic cell lines. The transient exposure of gestating females to vinclozolin during the fetal gonadal sex determination period promoted the epigenetic transgenerational inheritance of adult onset diseases in F1–F4-generation rats. The exposure to EDCs triggers epimutations in fetal germ cells that may be corrected in the next generation. The correction of epimutations aiming at preventing the transgenerational inheritance of such errors caused by environmental factors may have played an evolutionary role [97]. These transgenerational epigenetic changes found in sperm are likely exposure-specific. For instance, a parental transgenerational transmission of disorders via the female or male germline was suggested by using methoxychlor [104]. This observation indicates that these epimutations could be used as biomarkers for ancestral toxicant exposure. The imprinted genes methylation profile remains unchanged and thus is not reprogrammed, but parent-specific imprints are established during the epigenetic reprogramming process. The impact of exposure to EDCs is notable, particularly when tissues are differentiating and cells are sensitive to imprinting. A modification of any epigenetic marker impacts genetic programming, resulting in an alteration of the adult epigenome and transcriptome [143]. Somatic tissues can response to stressing stimuli by releasing EVs containing RNA. These vesicles may be internalized by epididymal spermatozoa, which in turn delivers the vesicles to oocytes at fertilization. Therefore, this process may be assimilated to a continuous stream of epigenetic information flows from parental somatic-tissue-derived embryos. The flow is capable of crossing the Weismann barrier, facilitated by circulating vesicles. This process ultimately results in the possibility to pass specific epigenetic traits on to the offspring [144].

## 6. Conclusions

Today, a growing number of studies indicate that environmental factors and particularly EDCs are transduced by both paternal and maternal gametes over the course of a pregnancy. Table 1 is a summary of studies that showed the transgenerational effect of exposure to EDCs. Better understanding the transgenerational effects of EDCs on reproductive health may help to predict their consequences on the next generations. However, the reality is more complex than all of the studies presented in this review. We must keep in mind that humans are exposed to EDC mixtures composed of hundreds of chemicals every day—not a single chemical in isolation. Improving our understanding of the epigenetic changes that occur in the parental germline following preconception environmental conditions and exposure will enhance reproductive success, as well as improve offspring health [145]. Numerous disruptions of the epigenome in mammals provoked by environmental exposure have been described. While some appear to be corrected by germline-specific epigenetic reprogramming, others remain uncorrected and are transmitted over subsequent generations [146]. There are recommendations to pregnant women who are vulnerable to EDCs: avoid products that have been in contact with pesticides, limit their consumption of fatty fish and crustaceans loaded with heavy metals and endocrine disruptors, avoid food plastics and above all do not heat them in the microwave, be careful with cosmetics by avoiding too many or by favoring organic products, ventilate housing regularly, and do not use stoves that have a non-stick coating. By changing some of these habits, it is possible to limit the cocktail of effects. The limitation of these substances become part of the awareness of consumers.

## Figures and Tables

**Table 1:** Studies showing a non-genetic effect across generations in different species.

Model	EDC	Transgenerational Effect	Reference
Daphnia magna microcrustacean	Flame retardants Tris(2-butoxyethyl) phosphate (TBOEP)	Levels of mRNA were found to be significantly different for genes known to be involved in endocrine-mediated mechanisms such as reproduction and growth between generations F0, F1, and F2, indicating the effects of parental exposure on offspring.	[89]
Crepidula onyx gastropod	2,2′,4,4′-tetrabromodiphenyl ether (BDE-47)	Bioaccumulation and maternal transfer of BDE-47 were evident in all life stages of the F0 generation and in F1 eggs, respectively. Exposure to BDE-47 reduced fecundity, delayed sexual maturity, and impeded embryonic development in F0 to F2.	[147]
Zebra fish (danio rerio)	Flame retardant Tetrabromobisphenol A (TBBPA)	Neurotoxicity and decreased content of dopamine in larval offspring.	[90]
Medaka	BPA EE2	BPA or EE2-induced transgenerational reproductive impairment in the F2 generation was associated with alterations in reproductive gene expression in brain and testis and global DNA methylation in testis.	[148]
Gobiocypris rarus	BPA	Parental BPA exposure inhibited the ovary development of the offspring.	[149]
Fish	Dioxin	Exposure to the environmental toxicants methylmercury or dioxin transmit to their grand-offspring behavioral changes, visual defects, increased body mass, skeletal abnormalities and/or decreased fertility, sometimes associated with changes in DNA methylation.	[150]
Medaka	BPA EE2	Medaka exposed to the endocrine disruptors BPA or ethinylestradiol produce grand-offspring and great-grand-offspring with reduced fertility.	[151]
Bird	Genistein	In quail eggs exposed to the environmental estrogen genistein, the great-grand offspring age at which the first egg was laid was significantly greater. Embryonic environment affects the phenotype of offspring three generations later in quail.	[152]
Rodent	Vinclozoline	Increased obesity risk in rats is inherited transgenerationally after ancestral exposure to DDT, plastic compounds, hydrocarbons and methoxychlor.	[130]
Rodent	Vinclozoline	Endocrine disruptors have been shown in mouse models to induce transmissible changes over several generations, altering the quality of spermatogenesis in adulthood.	[11,130]
Rodent	Chlordecone	Chlordecone increases prostatic epithelial neoplasia in F1 and F3 mice. Hoxa genes are affected both in the prostate and in sperm of F1 and F3 generations.	[24]
Fish M. beryllina	Bifenthrin (pyrethroid insecticide) Levonorgestrel (synthetic progestin), Ethinylestradiol (synthetic estrogen), Trenbolone (synthetic androgen)	Differential methylation of EDC-responsive genes is inherited by the offspring of EDC-treated animals, sometimes in the F2 generation that was never exposed. Low environmentally relevant levels of EDCs can cause altered methylation in genes that are functionally relevant to impaired phenotypes documented in EDC-exposed animals. EDC exposure has the potential to affect epigenetic regulation in future generations of fish that have never been exposed.	[8]
Zebrafish	TCDD (dioxin)	Multi- and transgenerational methylomic changes in testicular tissue and decreased reproductive capacity, significantly in the indirectly exposed F1 generation. Histone modification genes were both differentially methylated and expressed in all generations, and many differentially methylated genes overlapped between multiple generations.	[153]

## Data Availability

Not applicable.

## Abbreviations

AMH	Anti-Mullerian hormone
ART	Assisted reproductive techniques
BDE-47	2,2′,4,4′-tetrabromodiphenyl ether
BPA	Bisphenol A
DES	Diethylstilbestrol
DMRs	Differentially methylated regions
DDT	1,1,1-trichloro-2,2-bis(4-chlorophenyl)ethane
DEHP	Di(2-ethylhexyl) phthalate
EE2	Ethinylestradiol
EDCs	Endocrine-disrupting chemicals
Endo-siRNAs	Endogenous-small interfering RNAs
EVs	Extracellular vesicles
FDA	U.S. Food and Drug Administration
H3K9me3	H3K9 trimethylation
HAT	Histone acetyltransferase
HPTE	2,2-bis-(p-hydroxyphenyl)-1,1,1-trichloroethane
Igf2	Insulin-like growth factor 2
lincRNAs	Large intergenic non-coding RNAs
LIF	Leukemia inhibitory factor
lncRNA	Long non-coding RNA
miRNAs	MicroRNAs
mRNA	Messenger RNA
MXC	Methoxychlor (1,1,1-trichloro-2,2-bis(p-methoxyphenyl) ethane)
NOAEL	No-observed-adverse-effect level
OP	Organophosphate
OPFRs	Organophosphate flame retardants,
piRNAs	Piwi-interacting RNAs
PBDEs	Polybrominated diphenyl ethers
PAHs	Polycyclic aromatic hydrocarbons
PCBs	Polychlorinated biphenyls
PCOS	Polycystic ovary syndrome
PFOS	Perfluorooctane sulfonate
PFOA	Perfluorooctanoate
PGC	Primordial germ cells
PPAR	Peroxisome proliferator-activated receptor
snoRNAs	Small nucleolar RNAs
sncRNA	Small non-coding RNAs
SUV39H2	SUppressor of Variegation 3-9 homolog 2 Histone Lysine Methyltransferase
TBBPA	Tetrabromobisphenol A
TBT	Tributyltin
TCDD	2,3,7,8-tetrachlordibenzo-p-dioxin
TPT	Triphenyltin
Xist	X-inactive specific transcript
